# Neuronatin regulates pancreatic **β** cell insulin content and secretion

**DOI:** 10.1172/JCI120115

**Published:** 2018-07-09

**Authors:** Steven J. Millership, Gabriela Da Silva Xavier, Agharul I. Choudhury, Sergio Bertazzo, Pauline Chabosseau, Silvia M.A. Pedroni, Elaine E. Irvine, Alex Montoya, Peter Faull, William R. Taylor, Julie Kerr-Conte, Francois Pattou, Jorge Ferrer, Mark Christian, Rosalind M. John, Mathieu Latreille, Ming Liu, Guy A. Rutter, James Scott, Dominic J. Withers

**Affiliations:** 1 MRC London Institute of Medical Sciences, London, United Kingdom.; 2 Institute of Clinical Sciences, Faculty of Medicine, Imperial College London, London, United Kingdom.; 3 Section of Cell Biology and Functional Genomics, Division of Diabetes, Endocrinology and Metabolism, Department of Medicine, Imperial College London, London, United Kingdom.; 4 Department of Medical Physics and Biomedical Engineering, University College London, London, United Kingdom.; 5 Computational Cell and Molecular Biology Laboratory, Francis Crick Institute, London, United Kingdom.; 6 European Genomic Institute for Diabetes, UMR 1190 Translational Research for Diabetes, INSERM, CHU Lille, University of Lille, Lille, France.; 7 Beta Cell Genome Regulation Laboratory, Department of Medicine, Imperial College London, London, United Kingdom.; 8 Institute of Reproductive and Developmental Biology, Department of Surgery and Cancer, Imperial College London, London, United Kingdom.; 9 School of Biosciences, Cardiff University, Cardiff, United Kingdom.; 10 Department of Endocrinology and Metabolism, Tianjin Medical University General Hospital, Tianjin, China.; 11 National Heart and Lung Institute, Department of Medicine, Imperial College London, London, United Kingdom.

**Keywords:** Cell Biology, Genetics, Beta cells, Diabetes, Insulin

## Abstract

Neuronatin (*Nnat*) is an imprinted gene implicated in human obesity and widely expressed in neuroendocrine and metabolic tissues in a hormone- and nutrient-sensitive manner. However, its molecular and cellular functions and precise role in organismal physiology remain only partly defined. Here we demonstrate that mice lacking *Nnat* globally or specifically in β cells display impaired glucose-stimulated insulin secretion leading to defective glucose handling under conditions of nutrient excess. In contrast, we report no evidence for any feeding or body weight phenotypes in global *Nnat*-null mice. At the molecular level neuronatin augments insulin signal peptide cleavage by binding to the signal peptidase complex and facilitates translocation of the nascent preprohormone. Loss of neuronatin expression in β cells therefore reduces insulin content and blunts glucose-stimulated insulin secretion. *Nnat* expression, in turn, is glucose-regulated. This mechanism therefore represents a novel site of nutrient-sensitive control of β cell function and whole-animal glucose homeostasis. These data also suggest a potential wider role for *Nnat* in the regulation of metabolism through the modulation of peptide processing events.

## Introduction

Imprinted genes display monoallelic expression according to parent of origin due to epigenetic events initiated in the germline (1). Around 150 such genes have been identified in mice, 50% of which have also been found in humans. Although its origins remain obscure, mouse and human genetic studies have revealed key roles for genomic imprinting in regulating both development and various aspects of postnatal physiology. For example, many imprinted genes regulate placental function, fetal and neonatal growth, and early-life metabolism and behavior (2). Furthermore, a number of human syndromes with fetal and postnatal growth and metabolic phenotypes, such as Prader-Willi, Angelman, and Beckwith-Wiedemann syndromes, result from abnormalities in the expression of specific imprinted genes (2). Despite these advances, for the majority of imprinted genes, the precise cellular and molecular mechanisms through which their effects on growth and metabolism are mediated remain unclear.

Neuronatin (*Nnat*) is a paternally expressed imprinted proteolipid-encoding gene originally identified in the developing rat brain but also found in all placental mammals, including humans. It is expressed predominantly in the neuroendocrine systems of the developing fetus and adult but is also found in adipose tissue (3–5). The *Nnat* locus resides in a “microimprinted” region within the intronic sequence of the neighboring gene *Blcap*, with differential expression controlled by localized methylation of the silenced maternal allele (6–10). In vivo studies have shown that *Nnat* expression is dynamically regulated by nutrient status. For example, fasting and leptin administration down- and upregulate *Nnat* mRNA levels in the hypothalamus, respectively (11–13). Nutrient-dependent changes in expression are also seen in white adipose tissue and pancreatic β cells, and altered expression is found in the adipose tissue and islets of rodent models of obesity and diabetes (5, 14–16). Furthermore, recent evidence suggests that *Nnat* expression in mice, together with a cluster of other imprinted genes under the regulation of the chromatin-interacting protein TRIM28, underpins the “stochastic” development of obesity seen in inbred mouse strains (17). Single-nucleotide polymorphisms in the human *NNAT* locus are associated with extreme childhood obesity, and reduced *NNAT* expression has been reported in the adipose tissue of obese children (13, 17). Together, these findings suggest a role for neuronatin in the regulation of body weight and the pathophysiology of obesity, although the molecular mechanisms underlying these observations remain undetermined.

While to date no direct in vivo evidence has been provided for a role for *Nnat* in the regulation of glucose homeostasis, in vitro studies manipulating its expression in cultured pancreatic β cells suggest that *Nnat* may regulate glucose-stimulated insulin secretion (GSIS) (14, 18). In terms of the potential mechanisms underlying this observation, in vitro studies have shown that NNAT is present in the endoplasmic reticulum (ER) (14, 19) and, in part owing to a suggested structural resemblance to the calcium-handling protein phospholamban, have implicated this protein in the control of intracellular calcium dynamics (3, 5, 14, 20). Involvement in the control of ion channels, Ca^2+^ ATPases, and glucokinase has also been proposed (21).

Despite the above evidence implicating *Nnat* in the control of GSIS, a defined molecular and cellular mechanism that might underpin this role has yet to be defined. Furthermore, as no detailed characterization of an *Nnat*-deficient mouse model has previously been described, the precise in vivo functions of *Nnat*, with respect to pancreatic islet function and specifically to the distinct events regulating GSIS, remain unclear.

Here, we reveal that in vivo deletion of *Nnat* either globally or specifically in β cells causes defective GSIS, leading to impaired glucose tolerance under conditions of nutrient overload. Thus, *Nnat* is required for normal pancreatic β cell insulin secretion. In contrast, we find little evidence for any feeding or body weight phenotypes in global *Nnat*-null mice. We further demonstrate that NNAT binds to the signal peptidase complex (SPC) and facilitates the translocation of nascent preproinsulin into the ER. Impairment of preproinsulin signal peptide (SP) cleavage in β cells leads to a deficiency of available mature insulin secretory granules and defective GSIS. Neuronatin expression itself is regulated by glucose, and so, together, these studies identify a novel component of the insulin processing apparatus, illustrate the important contribution of the SPC to the molecular events involved in the control of GSIS, and thus define a previously unrecognized site of nutrient-sensitive control of β cell function and whole-animal glucose homeostasis. Our studies also suggest a potentially wider role for *Nnat* in the regulation of a range of peptide secretory processes relevant to growth and metabolism.

## Results

### Mice with targeted deletion of the Nnat gene display defective GSIS.

**Figure 1:**
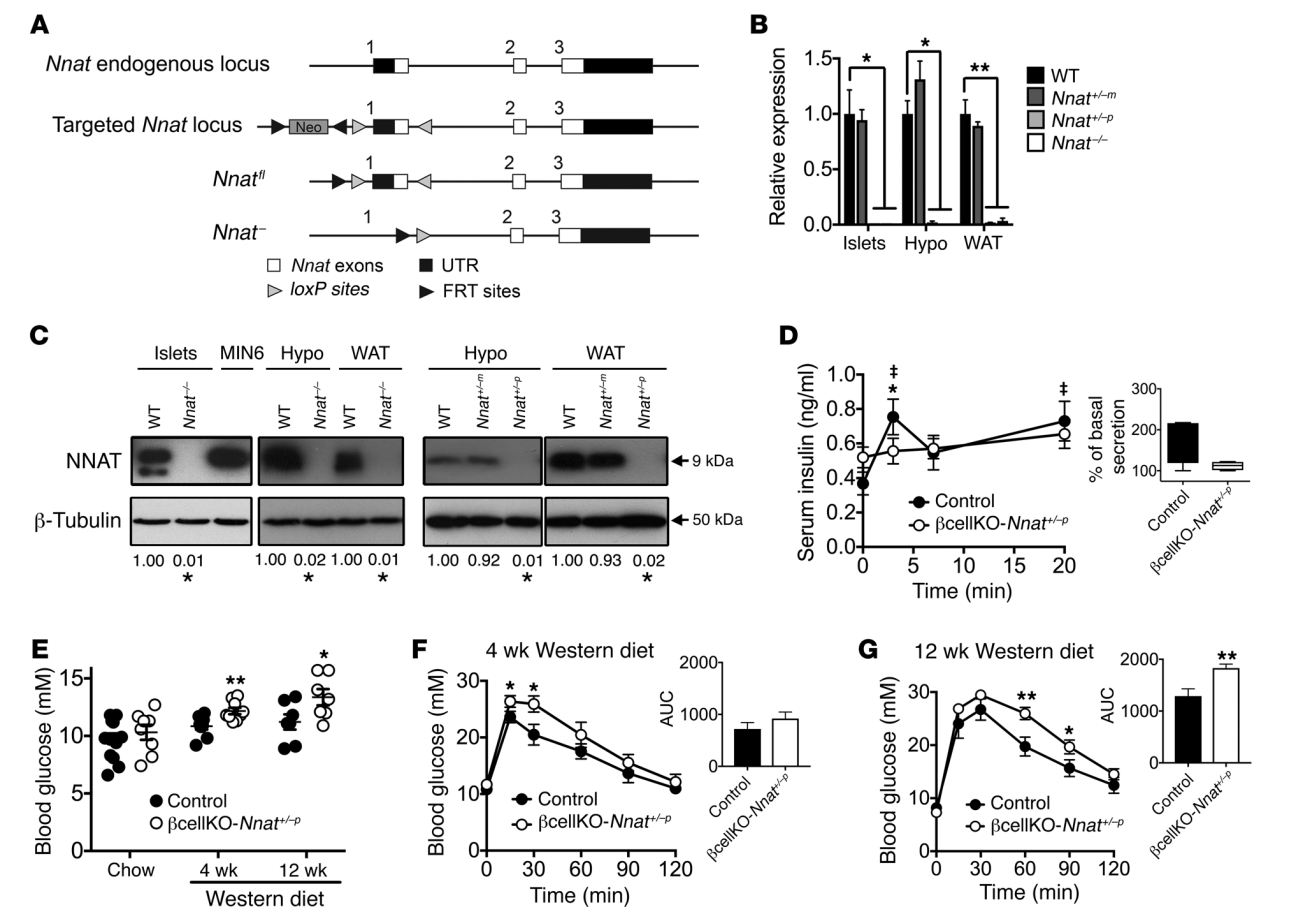
Effect of *Nnat* deficiency in vivo. (**A**) Targeted inactivation of the *Nnat* gene. Exon 1 was flanked by *loxP* sites with the *neomycin* selection cassette (Neo) flanked by FRT sites, to produce a floxed and null allele. (**B** and **C**) Quantitative RT-PCR and representative Western blot analysis of *Nnat* expression in tissues of WT, heterozygous *Nnat^+/–m^* (maternal deletion), heterozygous *Nnat^+/–p^* (paternal deletion), and homozygous *Nnat^–/–^* mice on C57BL/6J background. Data are compared with WT mice (*n* = 4–7 animals per group, Kruskal-Wallis or Mann-Whitney *U* test). (**D**) Measurement of insulin secretion in vivo in response to i.p. glucose in 10-week-old male βcellKO-*Nnat^+/–p^* versus control mice on C57BL/6J background (*n* = 8 animals per genotype, ANOVA with repeated measures). Inset shows box-and-whisker plot of the same data plotted as percentage insulin secretion across all time points compared with basal insulin values (at *T* = 0). (“‡” indicates statistically significant increases, *P* < 0.05, in secretion in WT mice compared with basal insulin values.) (**E**) Fasted (4-hour) blood glucose levels from 10-week-old chow-fed male βcellKO-*Nnat^+/–p^* versus control mice and from male mice of both genotypes fed Western diet for 4 weeks (14 weeks old) and 12 weeks (22 weeks old) (Student’s *t* test for each time point, all C57BL/6J, *n* = 7–14 animals per genotype, per time point, minimum 2 independent cohorts). (**F** and **G**) Glucose tolerance in overnight-fasted Western diet–fed groups as in **E** (ANOVA with repeated measures). Insets show means of area under the curve (AUC) for both genotypes at both time points (Student’s *t* test for each). (*P < 0.05, **P < 0.01).

To explore the role of *Nnat* in whole-body metabolism, we generated mice with global deletion of the *Nnat* gene (Figure 1A). A floxed *Nnat* allele (*Nnat^fl^*) was first created in embryonic stem cells by the introduction of *loxP* sites flanking exon 1. A globally *Nnat*-null mouse line (*Nnat^+/–^*) was then established following permanent excision of the flanked region by crossing of *Nnat^+/fl^* mice with germline *Cre* recombinase–expressing deleter mice. Globally null *Nnat^–/–^* animals backcrossed onto the C57BL/6J strain background were viable and fertile and were born with a normal Mendelian ratio with normal litter sizes. In adult mice, neuronatin is primarily expressed in adipose tissue, hypothalamus, pituitary, and pancreatic islet cells, and reverse transcription PCR (RT-PCR) and Western blotting analysis confirmed the absence of neuronatin expression in tissues of homozygous neuronatin-knockout (*Nnat^–/–^*) mice (Figure 1, B and C). Heterozygous mice receiving the mutant allele from the paternal side (*Nnat^+/–p^*) had undetectable levels of expression in islets, hypothalamus, and white adipose tissue (WAT) at the mRNA and protein level, indicating that the imprinting status of *Nnat* was maintained in the mutant animals. In contrast, heterozygous mice receiving the mutant allele from the maternal side (*Nnat^+/–m^*) displayed wild-type levels of *Nnat* expression (Figure 1, B and C). Targeting of *Nnat* did not affect expression of the biallelic gene *Blcap* found at the same locus (Supplemental Figure 1A; supplemental material available online with this article; https://doi.org/10.1172/JCI120115DS1).

In view of the existing evidence for a role for *Nnat* in energy homeostasis regulation, we assessed body weight and also feeding behavior (ad libitum, after fasting and in response to exogenous leptin). However, these were all unaltered in male *Nnat^+/–p^* mice compared with their WT littermates (Supplemental Figure 1, B–E). In contrast, when we analyzed glucose homeostasis we found that male *Nnat^+/–p^* mice displayed a defect in GSIS in vivo compared with WT littermates, with the complete absence of an increase from basal insulin levels, as well as unexpected basal hyperinsulinemia (Supplemental Figure 1F). This GSIS abnormality, which resulted in the loss of both phases of classical biphasic insulin secretion, was present in male knockout mice on both C57BL/6J and 129S2/Sv backgrounds (Supplemental Figure 1, F and G and insets).

**Figure 2:**
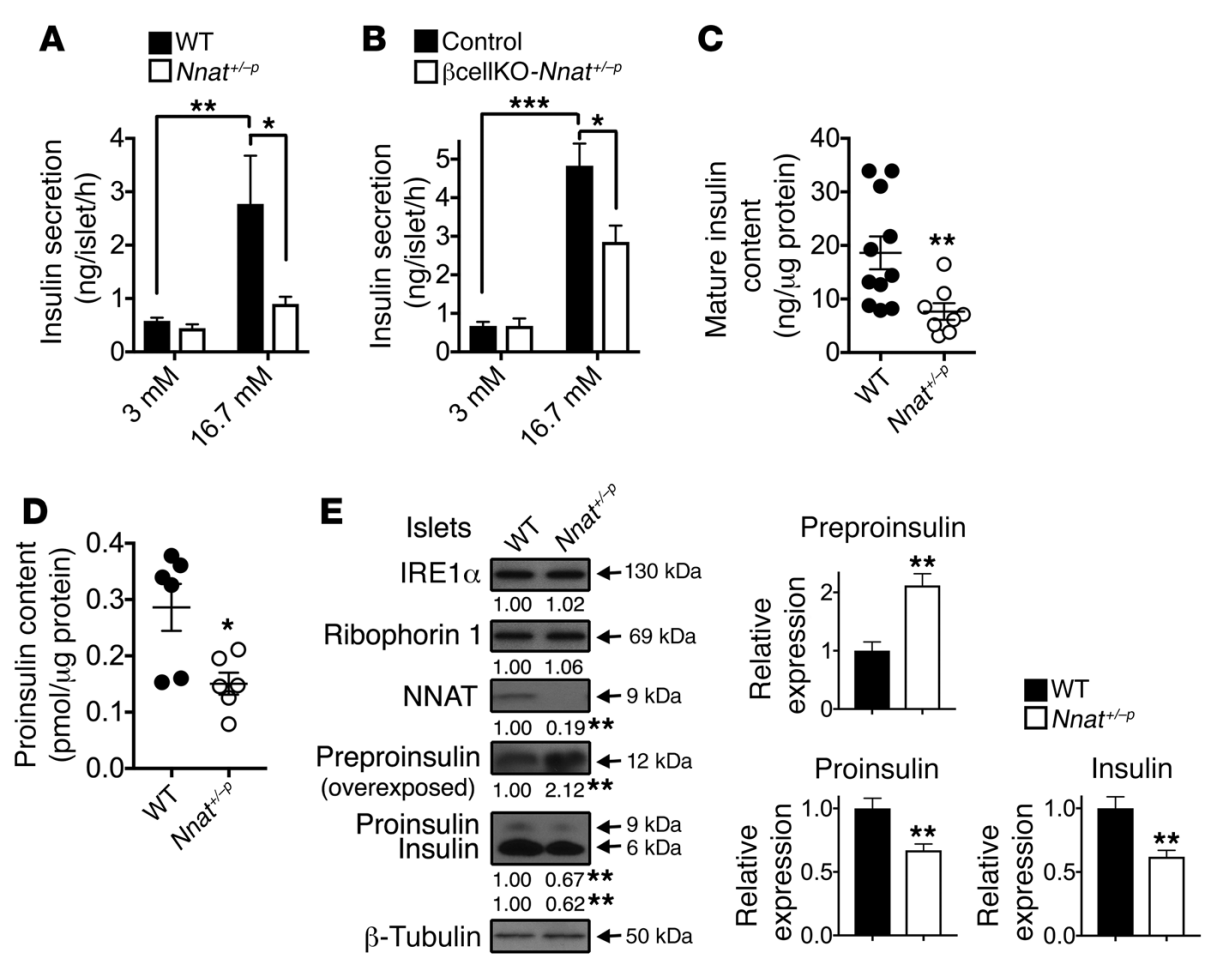
Insulin content and secretion in *Nnat*-deficient islets. (**A**) Insulin secretion in static incubations of primary isolated islets from 10-week-old male *Nnat^+/–p^* and WT mice was determined in vitro under low-glucose (3 mM) and high-glucose (16.7 mM) conditions (*n* = 12 animals per group, 2-way ANOVA). (**B**) Insulin secretion in static incubations of primary isolated islets from 10-week-old male control and βcellKO-*Nnat^+/–p^* mice was determined as in **A** (*n* = 8 mice per genotype, both 3 independent experiments). (**C** and **D**) Mature insulin content (**C**) (*n* = 11 for WT and 8 for *Nnat^+/–p^*, Student’s *t* test) and proinsulin content (**D**) (*n* = 6 animals per group, Mann-Whitney *U* test) were quantified in isolated islets from 10-week-old male WT and *Nnat^+/–p^* mice and normalized to total protein. (**E**) Western blotting analysis of protein levels in primary isolated islets from 10-week-old male *Nnat^+/–p^* and WT mice. A representative blot of 2 independent experiments (*n* = 4 mice per genotype, Student’s *t* test) is shown. β-Tubulin was used as a loading control. Mean values for band intensities in multiple experiments quantified by densitometry are shown below each panel as well as in associated bar charts for insulin species, all expressed relative to WT samples (*P < 0.05, **P < 0.01, ***P < 0.001).

To determine whether the perturbed GSIS seen in global *Nnat^+/–p^* mice was due to a β cell–intrinsic defect, and because islet neuronatin expression is almost exclusively restricted to β cells (Supplemental Figure 1H), we generated a β cell–specific knockout line by crossing our *Nnat^fl^* (floxed) line with mice harboring *Cre* recombinase knocked in at the *Ins1* locus. This deleter strain catalyzes efficient (~95%) recombination highly selectively in the β cell, with undetectable activity at extrapancreatic sites (22, 23). RT-PCR and Western blotting confirmed loss of *Nnat* expression specifically in pancreatic islets of heterozygous mice with a floxed *Nnat* allele from the paternal side (βcellKO-*Nnat^+/–p^*) but not from the maternal side (βcellKO-*Nnat^+/–m^*) and with no alterations in *Blcap* expression (Supplemental Figure 1, I–K). While WT, *Cre*-positive, floxed, and βcellKO-*Nnat^+/–m^* mice all had normal GSIS (and were combined as controls), this was essentially abolished in male βcellKO-*Nnat^+/–p^* mice (Figure 1D), indicating that the GSIS phenotype is β cell–autonomous. When exposed to a Western diet for 12 weeks, control mice retained biphasic GSIS (Supplemental Figure 1L). In contrast, βcellKO-*Nnat^+/–p^* mice did not display a normal hyperinsulinemic response to overnutrition, with persistence of the defect in GSIS previously seen on chow diet (control: 3.5-fold increase in basal insulin levels with Western diet vs. 2.2-fold in βcellKO-*Nnat^+/–p^* mice, *n* = 7 per genotype, *P* = 0.0021; Supplemental Figure 1L and inset). Fasting blood glucose, which was unaffected in chow-fed mice, progressively increased in βcellKO-*Nnat^+/–p^* animals compared with controls during exposure to Western diet (Figure 1E). Glucose tolerance was unaffected by *Nnat* deletion in chow-fed mice (Supplemental Figure 1M). However, a progressive deterioration in glucose tolerance was seen over a 12-week period in male βcellKO-*Nnat^+/–p^* mice on the Western diet (Figure 1, F and G). No differences in body weight or insulin sensitivity were apparent between groups (Supplemental Figure 1, N and O), suggesting a progressive decline in β cell function rather than insulin action in mutant mice with overnutrition. This view was further supported by in vitro studies using islets isolated from both global *Nnat^+/–p^* and conditional βcellKO-*Nnat^+/–p^* mice on a C57BL/6J background, which revealed a marked impairment in GSIS in the mutant β cells (global KO: 4.8-fold insulin secretion increase with high glucose in WT vs. 2.0-fold in *Nnat^+/–p^*, *n* = 12, *P* = 0.0401; β cell conditional KO: 7.2-fold increase in controls vs. 4.2-fold in βcellKO-*Nnat^+/–p^*, *n* = 8, *P* = 0.0207; Figure 2, A and B, respectively). Taken together these findings indicate that in vivo, *Nnat* regulates β cell function but has no effect on body weight and feeding behavior.

### Nnat-deficient primary β cells have a defect in insulin storage.

Next, we explored a number of cellular and molecular mechanisms potentially responsible for defective GSIS in *Nnat*-mutant animals. Impairment of GSIS on a chow diet was not due to a reduction in β cell mass or disruption of islet architecture (Supplemental Figure 2, A and B). Furthermore, there was no reduction in β cell mass in βcellKO-*Nnat^+/–p^* mice following Western diet feeding (control: 1.74 ± 0.36 mg vs. 1.49 ± 0.30 mg in βcellKO-*Nnat^+/–p^* mice, *n* = 5 per genotype, *P* = 0.5476), suggesting that the deficit in GSIS on this diet was not due to loss of β cells. The expression of key markers of the mature β cell, including *Glut2* (*Slc2a2*), *Neurod1*, *Ins2*, *Pdx1*, and *Nkx6.1*, was unaltered in islets from global *Nnat*-knockout animals, as were serum glucagon levels (Supplemental Figure 2, C and D).

Manipulating expression of NNAT in β cells and other cell types has previously been demonstrated to perturb intracellular Ca^2+^ homeostasis and dynamics, and it has been suggested that this underlies the defect in GSIS found after in vitro knockdown of *Nnat* (5, 14, 20). In contrast, we obtained no evidence for altered Ca^2+^ signaling in islets from *Nnat^+/–p^* mice in response to either Ca^2+^ influx provoked by high glucose, or release of Ca^2+^ from intracellular stores in response to the activation of G_q_-coupled muscarinic receptors, and the opening of ER-resident inositol (1,4,5)-*tris*phosphate receptors (Supplemental Figure 2E).

**Figure 3:**
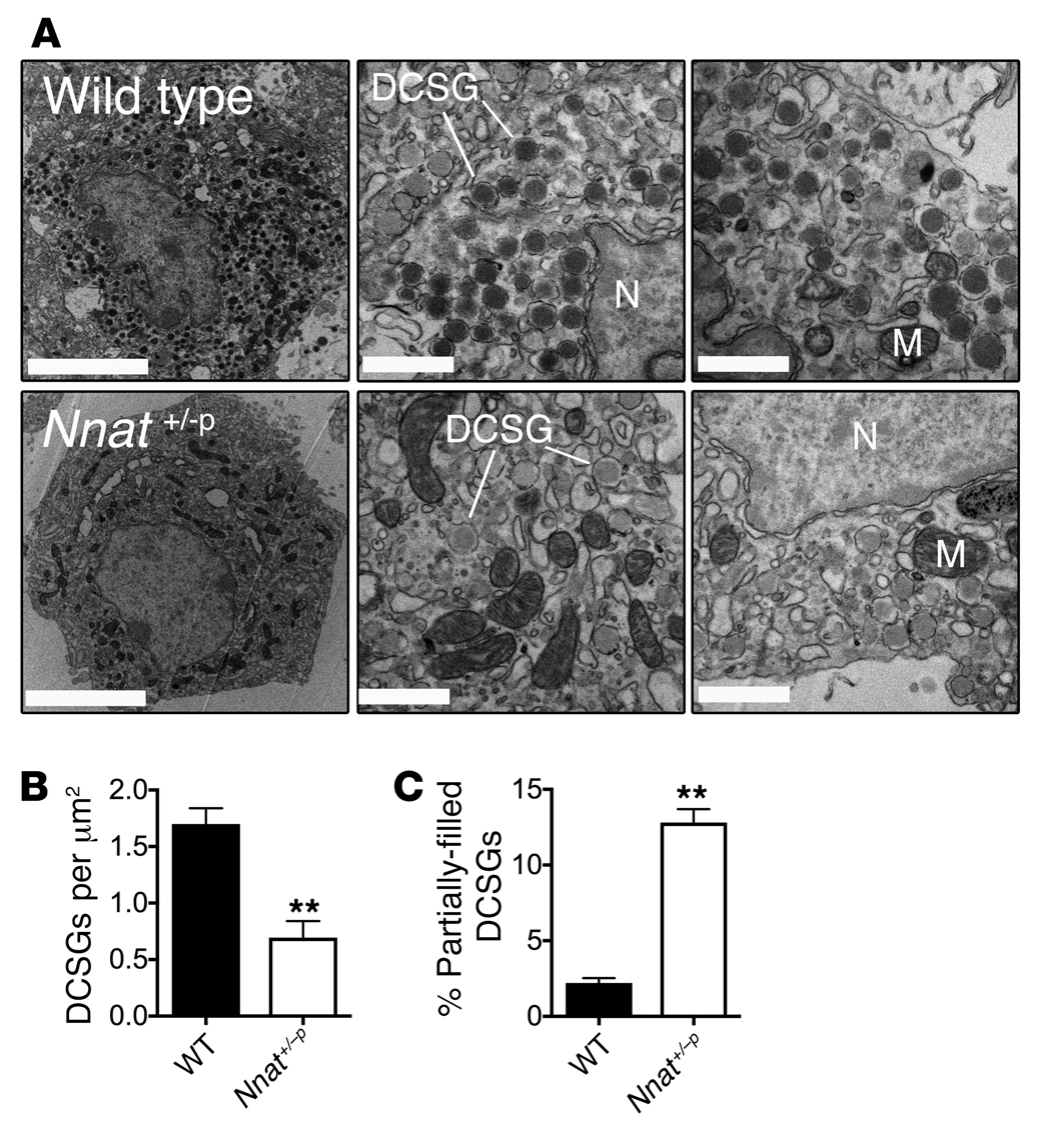
Insulin secretory granule morphology in *Nnat*-deficient β cells by electron microscopy. (**A**) Left: Representative electron micrographs of β cells from 10-week-old male WT and *Nnat^+/–p^* mice in ultrathin sections (scale bars: 5 μm). Middle and right: Higher-magnification images showing dense core secretory granules (DCSG), nuclei (N), and various mitochondria (M) (scale bars: 1 μm). A total of 9 β cells from sections of fixed islets were analyzed from 3 different animals per genotype. (**B** and **C**) Quantification of the number of DCSGs per unit area, and also percentage of partially filled DCSGs, using electron microscopy images from WT and *Nnat^+/–p^* β cells in **A** (**P < 0.01, both Student’s *t* test).

To determine whether defective GSIS in *Nnat*-deficient mice was due to a shortage of available cellular insulin, we measured mature insulin content in mutant primary islets by ELISA and found that it was significantly reduced compared with that in WT islets, potentially indicating a defect in insulin storage (Figure 2C). We also found a similar reduction of proinsulin content in islets from mutant mice, and therefore the proinsulin/mature insulin ratio was unaltered (Figure 2D and Supplemental Figure 2F). When primary islets from *Nnat^+/–p^* mice were treated with the proteasome inhibitor MG132 to prevent degradation of insulin precursors, Western blotting of lysates confirmed reduced proinsulin and mature insulin content in islets from mutant mice, and also the accumulation of unprocessed preproinsulin (Figure 2E). In contrast, no reduction in expression was observed for 2 other, ER membrane, proteins, ribophorin 1 and IRE1α (Figure 2E). The defect in insulin storage was confirmed by transmission electron microscopy, with *Nnat^+/–p^* β cells showing a reduction of mature insulin–containing dense core secretory granules, and an increased proportion of partially filled, or even “empty” (electron-translucent), granules (Figure 3, A–C). Together, these observations suggest a key in vivo role for *Nnat* in maintaining sufficient insulin content in pancreatic β cells for normal GSIS.

### Neuronatin interacts with the SPC.

**Figure 4:**
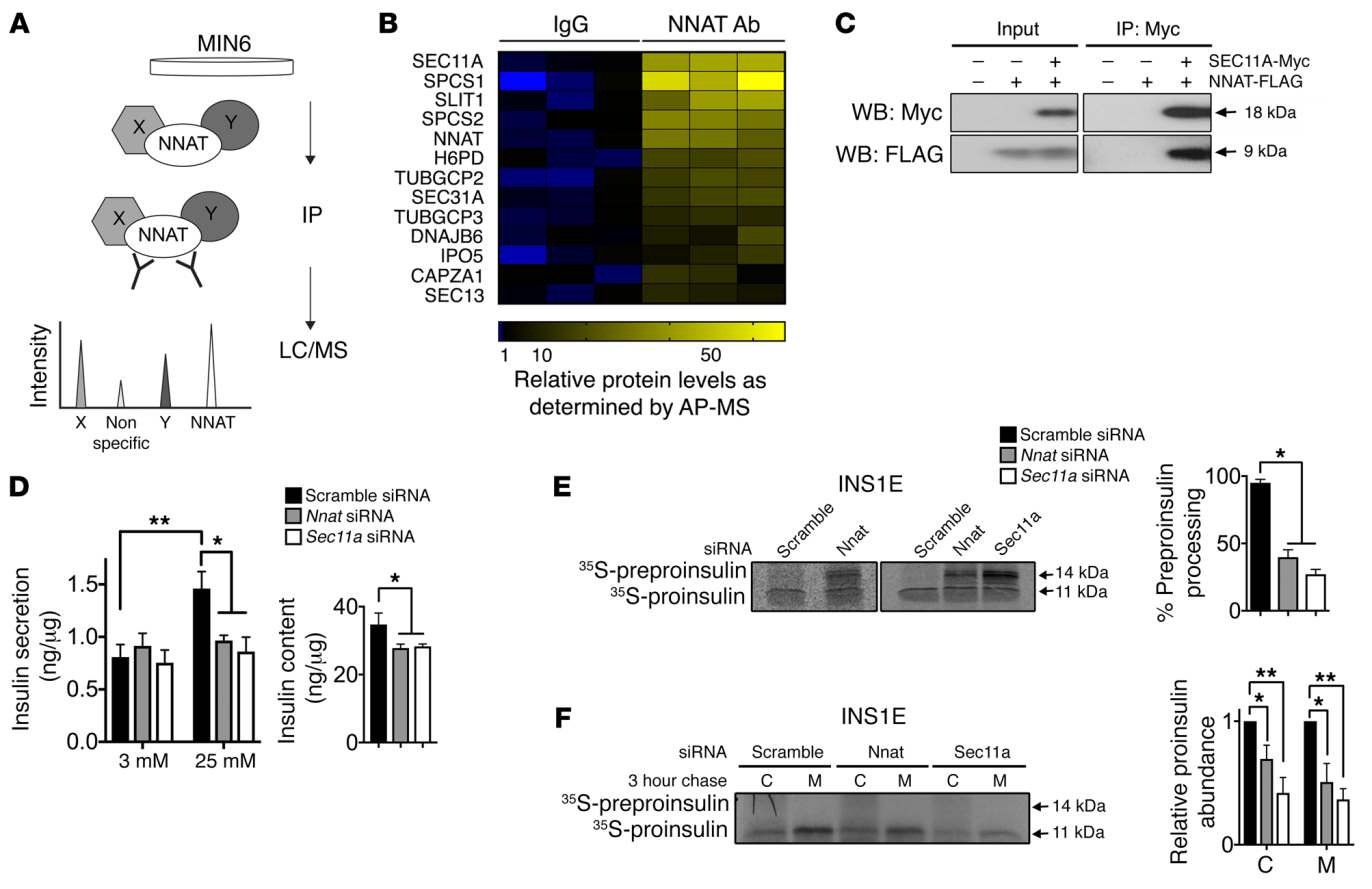
NNAT interaction with the SPC and modulation of preproinsulin handling. (**A**) Overview of affinity purification/mass spectrometry (AP/MS) screen for novel interaction partners of NNAT. Endogenous NNAT was immunoprecipitated (IP) from MIN6 cell lysates and interacting partners in co-IPs analyzed by liquid chromatography/mass spectrometry (LC/MS). (**B**) Heatmap from AP/MS analysis of top protein hits in IPs using antibodies against NNAT (NNAT Ab) and control IPs with rabbit immunoglobulins (IgG). Relatively high abundance is shown in yellow and relatively low abundance in blue. (**C**) Lysates from HEK293T cells expressing c-Myc–tagged SEC11A and FLAG-tagged NNAT were immunoprecipitated using anti–c-Myc antibodies. Proteins in input and IP samples were detected by Western blotting using anti–c-Myc and anti-FLAG antibodies. Panel shows a representative blot of 3 independent experiments. (**D**) INS1E cells transiently transfected with siRNA targeting *Nnat* or *Sec11a* were assayed in vitro for GSIS at low (3 mM) and high (25 mM) glucose. A scramble siRNA served as a control with data expressed as mean insulin secretion per unit cellular protein. Graph on the right shows total insulin content in cell lysates. (*n* = 9 independent cultures per group, 3 independent experiments, 2-way ANOVA [left graph] and 1-way ANOVA [right graph].) (**E**) INS1E cells transfected with c-Myc–tagged preproinsulin and siRNAs targeting *Nnat* or *Sec11a* were pulse-labeled with ^35^S-Cys/Met. Lysates immunoprecipitated with anti–c-Myc agarose were analyzed by autoradiography. Associated bar chart shows preproinsulin and proinsulin band intensities in multiple experiments quantified by densitometry and expressed as percentage processing of preproinsulin (*n* = 3 cultures per group from 3 independent experiments, 1-way ANOVA). (**F**) Similar experiments performed as in **E**, from 3-hour chase cell lysates (C) and media (M), quantified as in **E** (*n* = 4 cultures per group from 3 independent experiments, 1-way ANOVA for both C and M). (*P < 0.05, **P < 0.01).

To gain further insights into the molecular roles of neuronatin, and provide potential mechanisms for our in vivo observations above, we used an unbiased affinity purification/mass spectrometry screen with the aim of identifying novel interaction partners of NNAT. We chose to focus on clonal pancreatic β cells in our proteomic approach to avoid issues of cellular heterogeneity from whole pancreatic islets. Protein lysates from MIN6 cells, a mouse insulinoma-derived β cell line (24), were incubated with antibodies against endogenous NNAT, and the immunoprecipitate analyzed by mass spectrometry (Figure 4A and Supplemental Figure 3A). Proteins coimmunoprecipitating with NNAT were ranked according to their abundance compared with control immunoprecipitates. Strikingly, of the top-ranking proteins in this analysis, 3 were components of the signal peptidase complex (SPC), namely the catalytic subunit SEC11A and signal peptidase complex subunits 1 and 2 (SPCS1 and SPCS2; Figure 4B and Supplemental Table 1 for full list), a complex responsible for cleavage of the signal peptide (SP) from nascent preprohormones. To verify the interaction between NNAT and the SPC, tagged versions of SEC11A and NNAT were expressed in cultured cells. FLAG-tagged NNAT coimmunoprecipitated with c-Myc–tagged SEC11A, confirming the association between NNAT and the SPC (Figure 4C).

### Neuronatin enhances signal peptidase activity in vitro.

The role of the SPC in the SP cleavage of preproinsulin has, in part, been established through the identification of human *insulin* gene mutations that alter preprohormone processing. This, in turn, impairs β cell function, leading to a spectrum of diabetic phenotypes (25, 26). siRNA-mediated transient silencing of *Sec11a* in the INS1E β cell line (27) reduced both insulin content and secretion in high-glucose conditions (Figure 4D and Supplemental Figure 3, B and C), suggesting a key role of the SPC in insulin maturation and subsequent release. Likewise, knockdown of *Nnat* caused a reduction in insulin content and marked blunting of GSIS (Figure 4D and Supplemental Figure 3, B and C).

To determine whether there is a direct role for NNAT in preproinsulin SP cleavage, we followed this event in newly synthesized preprohormone by pulse-labeling with ^35^S-methionine/cysteine. In cells transfected with a scrambled siRNA control, preproinsulin was rapidly processed to proinsulin and subsequently secreted (Figure 4, E and F). In contrast, *Nnat* silencing reduced preproinsulin SP cleavage, with significant preproinsulin accumulation (Figure 4E). Following a 3-hour chase period, we observed reduced secretion of proinsulin as well as some intracellular proinsulin loss, presumably reflecting degradation (Figure 4F). An even more marked defect in SP cleavage and secretion was observed upon knockdown of *Sec11a* (Figure 4, E and F). Together, these data suggest that NNAT interacts with, and modulates, preproinsulin processing by the SPC.

### NNAT resides on the cytosolic face of the ER membrane to regulate preproinsulin translocation.

**Figure 5:**
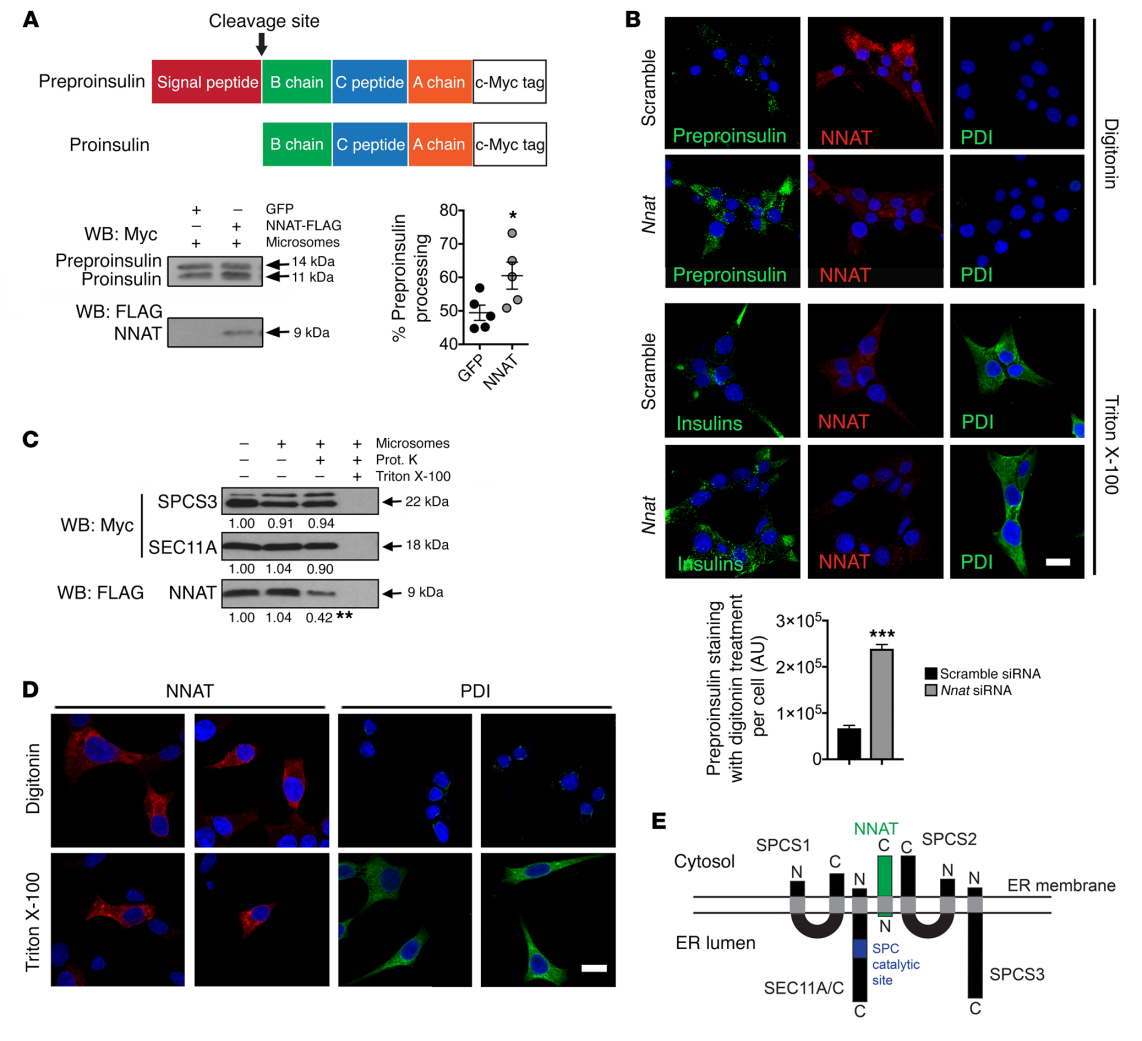
ER membrane topology of NNAT and its direct effect on SPC processing. (**A**) Representative Western blotting analysis of in vitro–translated preproinsulin converted to proinsulin in the presence (+) of pancreatic microsomes with and without coexpression of NNAT, expressed as percentage processing of preproinsulin. Coexpression of GFP was used as a control (*n* = 5 reactions per group, *P < 0.05, Mann-Whitney *U* test). (**B**) INS1E cells with *Nnat* siRNA knockdown versus scramble siRNA control were permeabilized with digitonin or Triton X-100, immunostained using an antibody that detects all insulin species (Insulins, green) and also NNAT (red), and visualized by confocal microscopy. The luminal ER protein PDI (green) was used to assess membrane permeabilization, and nuclei were visualized with DAPI. Scale bar: 10 μm. Fields of view were quantified for total fluorescence using ImageJ (NIH) from insulin-stained cells permeabilized with digitonin and normalized to cell number (Student’s *t* test, ***P < 0.001). (**C**) Representative Western blotting analysis of C-terminal c-Myc–tagged SPCS3 and SEC11A, and FLAG-tagged NNAT translated in vitro in the presence (+) or absence (–) of pancreatic microsomes and treated with proteinase K (Prot. K) (*n* = 3 reactions per group, mean vs. absence of microsomes, **P < 0.01, Student’s *t* test vs. presence of microsomes). (**D**) Immunofluorescent staining of INS1E cells permeabilized with digitonin or Triton X-100 with use of antibodies against NNAT (red) and PDI (green) visualized by confocal microscopy. PDI was used to assess membrane permeabilization, and nuclei were visualized with DAPI. Scale bar: 10 μm. (**E**) Topology of NNAT (green) and subunits of the SPC (black) on the ER membrane. The catalytic site for signal peptidase cleavage in SEC11A/C is shown in blue (N and C, amino- and carboxy terminal, respectively).

To explore the possibility of a direct effect of NNAT on signal peptidase activity, we used an in vitro translation system in which preproinsulin was synthesized in the presence of pancreatic microsomes, ER membrane fragments containing endogenous signal peptidase (and glycosylation) activity (28, 29). Initially, we confirmed that the microsomal membranes were able to support proteolytic cleavage of c-Myc–tagged preproinsulin, yielding an 11-kDa fragment corresponding to SP-cleaved proinsulin (Supplemental Figure 4A). Translation assays involving coexpression of c-Myc–tagged preproinsulin and either FLAG-tagged NNAT or GFP were then performed, with each reaction including a fixed concentration of microsomal membranes as a source of SPC. This approach demonstrated that coexpression of NNAT caused a small but significant augmentation of the SP processing of newly synthesized preproinsulin (Figure 5A) and suggested a key contribution for NNAT in the efficient functioning of the SPC.

Improper processing of preproinsulin might reflect reduced enzymatic activity of the SPC and/or failure of efficient preproprotein translocation across the ER membrane to the luminal surface where catalytic activity resides. To dissect the potential contribution of NNAT to each process, we first investigated whether preproinsulin targeting to the ER was perturbed in the absence of NNAT. Clonal INS1E β cells silenced for *Nnat* by RNAi were radiolabeled as above and treated with digitonin to specifically permeabilize plasma membranes, leaving internal (ER, secretory granule) membranes intact. Treatment of cells with digitonin, and subsequent centrifugation, led to a shift of GAPDH from the pellet to the supernatant, whereas insulin precursors were retained in the pellet in both control and *Nnat*-silenced cells. Thus, association of nascent preproinsulin with the ER is not perturbed upon silencing of NNAT (Supplemental Figure 4B). This observation indicates that, in the absence of NNAT, preproinsulin is still efficiently targeted to the ER membrane. However, it does not exclude the possibility that preproinsulin is not fully translocated across the ER membrane. To test this, we examined endogenous preproinsulin distribution by immunofluorescence in digitonin-treated, and thus partially permeabilized, cells, using an antibody that detects preproinsulin, proinsulin, and mature insulin. Cells were pretreated with MG132 to inhibit the action of the proteasome. Preproinsulin staining in digitonin-treated cells was only detected upon RNAi silencing of NNAT, suggesting that a portion of preproinsulin was untranslocated and had accumulated in the cytosol (Figure 5B). In control cells this observation was virtually absent, indicating complete translocation into the ER (Figure 5B). Staining of mature insulin, which was sequestered in secretory granules, was only present upon permeabilization of all membranes using Triton X-100 (Figure 5B). The ER luminal protein protein disulfide isomerase (PDI), which was used to monitor permeabilization, was also only stained after Triton X-100 permeabilization, in parallel cultures (Figure 5B). Together, these data demonstrate that, in the absence of NNAT, preproinsulin is still targeted to the ER but exhibits impaired translocation across the ER membrane.

Three of the five SPC subunits reside on the luminal side of the ER membrane (SEC11A, SEC11C, SPCS3), including both catalytic subunits, with the bulk of the remaining 2 proteins lying on the cytosolic side (SPCS1 and SPCS2) (30, 31). NNAT is a membrane-resident protein (Supplemental Figure 4C), with a proposed transmembrane region at the N-terminus. However, the localization of the C-terminal region has not been determined. To interrogate the topology of the SPC interaction with NNAT, we used in vitro translation to express C-terminally tagged NNAT on microsomal membranes and assessed susceptibility to proteinase K treatment. C-terminally tagged SPCS3 and SEC11A — a region of each protein known to be luminal (31) and therefore on the inside of the microsome — were used as controls. Translation of NNAT in the presence of microsomes did not alter its molecular weight, suggesting that NNAT itself is not cleaved or glycosylated (Figure 5C). Levels of the glycosylated form of SPCS3, represented by the upper band in Figure 5C, was increased in the presence of microsomes (31). Although the amount of SPCS3 and SEC11A protein was not altered by proteinase K treatment, presumably reflecting sequestration within microsomes, NNAT protein levels were substantially reduced in the presence of the protease, indicating that its C-terminal region, and indeed the bulk of this protein, is located on the cytosolic side of the ER membrane (Figure 5C). We further verified this observation in clonal INS1E β cells by fixing and permeabilizing cells with digitonin and immunostaining for endogenous NNAT with an antibody that recognizes the C-terminal domain. Digitonin treatment was sufficient to allow staining of NNAT, but not PDI, which was only stained after permeabilization of all membranes using Triton X-100 (Figure 5D). Together, these experiments demonstrate that the bulk of the NNAT protein and its C-terminus is located on the cytosolic side of the ER membrane, with an N-terminal anchor sequence (Figure 5E).

### Nnat expression is glucose-sensitive in rodent β cells.

**Figure 6:**
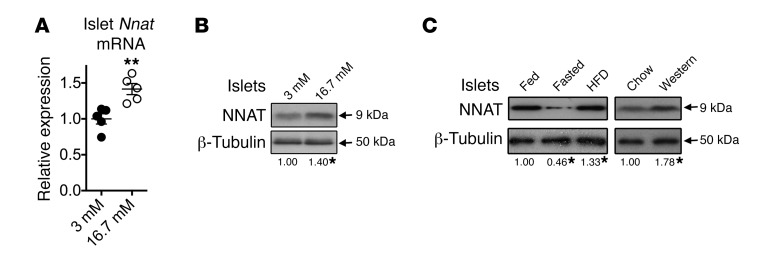
Regulation of NNAT expression in islets by glucose and diet. (**A**) Quantitative RT-PCR analysis of *Nnat* mRNA in isolated islets from 10-week-old male WT C57BL/6J mice cultured in low-glucose (3 mM) or high-glucose (16.7 mM) conditions for 6 hours. *Hprt* mRNA was used as an internal control, and data are compared with 3-mM cultures (*n* = 5 animals per group, Mann-Whitney *U* test). (**B**) Parallel islet preparations receiving the same treatment as in **A** were analyzed for protein expression by Western blotting. β-Tubulin was used as a loading control. (**C**) Representative Western blot analysis of NNAT protein expression in isolated pancreatic islets of 10-week-old male WT C57BL/6J mice that were chow-fed (Fed), fasted overnight (Fasted), or fed high-fat diet for 72 hours (HFD). Similar experiments were also performed with 72-hour feeding with Western diet (Western) compared with chow-fed controls (Chow). β-Tubulin was used as a loading control (*n* = 5 animals per group, Kruskal-Wallis for HFD studies, left panels, and Mann-Whitney *U* test for Western diet studies, right panels). Mean values for each condition are shown below each panel, compared with chow-fed controls. (*P < 0.05, **P < 0.01).

We (11, 15) and others (12, 13) have previously shown that *Nnat* expression is responsive to nutritional status in adipose tissue and hypothalamus. In view of the important role of NNAT in insulin SP cleavage and GSIS (Figures 1–5), we further examined the regulation of *Nnat* expression by glucose. In the present studies, acute (6-hour) incubation of isolated mouse islets (Figure 6, A and B) or clonal INS1E β cells (Supplemental Figure 5A) with high (16.7 mM) glucose caused rapid upregulation of *Nnat* mRNA and protein. Fasting in mice resulted in reduced NNAT protein levels in pancreatic islets, while acute feeding with either high-fat diet or Western diet led to increased expression (Figure 6C and Supplemental Figure 5B). Changes with similar dynamics were observed in adipose tissue (Supplemental Figure 5, C and D) and hypothalamus (Supplemental Figure 5E) from the same animals. Together, it appears that changes in the cellular levels of NNAT in response to nutritional cues, notably glucose, coordinately regulate subsequent alterations in demand for insulin SP cleavage by fluctuating glucose levels.

## Discussion

The present studies demonstrate a key and novel role for the nutrient-sensitive, imprinted gene neuronatin in the regulation of pancreatic β cell function. Through a series of in vitro and in vivo approaches, we show that NNAT directly interacts with the SPC to regulate the efficiency of preproinsulin signal peptidase cleavage. Neuronatin expression itself is glucose-regulated, thus revealing a previously unrecognized point of metabolic control for early maturation of insulin and therefore its storage and secretion. Loss of neuronatin function thus results in a defect in GSIS both in vitro and in the living animal.

The majority of secreted proteins are synthesized with an amino-terminal SP, directing them into the secretory pathway via the ER. Here, the membrane-bound SPC cleaves the SP, an essential step in the maturation and eventual secretion of the mature protein (32, 33). In mammals, the SPC consists of 5 subunits: SEC11A and SEC11C, which are responsible for the catalytic activity and are present in the ER lumen (31, 34, 35); and SPCS1, SPCS2, and SPCS3. SPCS1 and SPCS2, which are found on the cytosolic face, appear to affect proteolytic efficiency without possessing intrinsic catalytic activity, possibly by mediating preproprotein translocation across the ER membrane (30, 36–39). We show that in pancreatic β cells NNAT is located on the cytosolic face of the ER, presumably interacting with SPCS1 and SPCS2, where it promotes translocation but not targeting of nascent proinsulin into the ER. In contrast, we obtained little evidence for localization to the plasma membrane as suggested by others (21).

Our loss-of-function studies demonstrate that NNAT is required for efficient SP cleavage of preproinsulin by the SPC in β cells. Thus, disruption of this step results in reduced intracellular proinsulin and would appear, therefore, to lead to a defect in mature insulin storage and secretion, with mice lacking *Nnat* either globally or specifically in β cells displaying impaired GSIS. Interestingly, we have been unable to find previous published evidence to suggest that this early stage of insulin processing by the SPC is subject to specific regulation, and, consequently, it has been assumed that this step is constitutive in nature. Our RNAi studies in the INS1E β cell line demonstrate the importance of adequate SPC activity for insulin maturation and that early defects are able to propagate through the system and result in blunted GSIS. Furthermore, a key role for appropriate cleavage of the insulin SP in normal glucose homeostasis has previously been revealed by the identification of rare human genetic mutations within the SP region of preproinsulin (26). These mutations disrupt SP cleavage, leading to reduced insulin secretion in a dominant fashion, resulting in ER stress and β cell apoptosis, in a form of diabetes termed mutant insulin gene–induced diabetes of youth (MIDY) (25, 26, 40, 41). The magnitude of this β cell stress from mutant insulin species dictates the outcome of severe/early-onset or mild/late-onset diabetes. For example, an R6C mutation causes an approximately 50% reduction in translocation into the ER, with untranslocated molecules degraded in the cytosol. Successfully translocated preproinsulin is efficiently processed and secreted, with no evidence for ER stress, resulting in a milder form of mid- to late-onset diabetes (40, 42, 43). In contrast, an A24D insulin mutation does not affect preproinsulin translocation but completely blocks SP cleavage by SPC in the ER lumen, leading to misfolding and accumulation of unprocessed preproinsulin and subsequent ER stress, β cell failure, and neonatal diabetes (41, 44). We did not observe any evidence of ER stress in islets after *Nnat* inactivation in vivo (data not shown), with in vitro studies indicating a defect in preproprotein translocation on the cytosolic side of the ER membrane, where the bulk of the NNAT protein resides. Pulse-chase and immunofluorescence analysis further suggested that untranslocated preproinsulin is degraded in the cytosol. Consistent with this lack of ER stress, the proinsulin/mature insulin ratio in mutant islets was unaltered, indicating that processing of the C-peptide, downstream of the SP processing event cleavage and mediated by prohormone convertases (PCSK1 and PCSK2) and carboxypeptidase E (CPE), was unaffected. It is therefore clear that loss of neuronatin does not cause an absolute block in preproinsulin SP cleavage, as can occur in severe forms of MIDY. This may explain the lack of development of progressive diabetes in *Nnat*-deficient mice despite the presence of a severe defect in GSIS and glucose intolerance under nutrient overload, due to the absence of ER stress and subsequent β cell failure that can result from the accumulation of misfolded insulin species.

Instead, β cell *Nnat* deficiency in mice causes loss of appropriate insulin secretion control, with complete absence of glucose-evoked insulin release and glucose intolerance under conditions of nutrient excess. The mildly increased basal insulin secretion observed in vivo, but not in primary islets, likely stems from compensatory responses to lack of GSIS during periods of increased demand, i.e., feeding. This is perhaps the reason, together with the lack of a destructive islet phenotype and preservation of β cell mass, for the absence of glucose intolerance in chow-fed β cell–specific *Nnat*-deficient mice. However, this compensatory basal hyperinsulinemia was lost when these mutant mice were chronically challenged with a Western diet. Moreover, unlike control animals, the mutant mice failed to adequately increase basal insulin levels upon overnutrition, resulting in hyperglycemia and progressive glucose intolerance when exposed to this challenge. This abnormality was not due to a reduction in β cell mass on this diet, further suggesting a functional deficit in insulin production and release. The development of hyperglycemia may, in turn, propagate the defect in islet function through glucotoxicity, although our data do not suggest that this is an initiating event underlying the defective GSIS. We also observe that NNAT expression in β cells is rapidly responsive to acute changes in nutrient conditions both in vitro and in vivo and therefore appears to regulate β cell function in response to overnutrition.

Our studies also provide important insights into the previously proposed mechanisms through which neuronatin regulates cell physiology. Overexpression studies in vitro in both β cell and adipocyte cell lines had suggested that neuronatin regulates intracellular calcium handling (5, 14). In contrast, the present detailed studies of Ca^2+^ dynamics in *Nnat*-null primary β cells failed to reveal any defects. These findings strongly suggest that neuronatin is unlikely to be involved in shaping Ca^2+^ signals in these cells and specifically in the proximal calcium-dependent events underpinning glucose sensing (45). Furthermore, it does not appear that NNAT is present on insulin secretory granules itself. The role of NNAT in regulating SP cleavage and the ER localization of NNAT and its binding partners therefore point to a major role during initial peptide handling rather than other events involved in GSIS. While our studies cannot categorically exclude roles for neuronatin in other components of the complex processes by which β cells synthesize and secrete insulin in response to glucose (indeed we find that neuronatin associates with components of the COPII machinery), we believe that defects in SP processing and subsequent granule loading are likely to account for the major part of the observed defect in β cell function. Indeed, it should also be noted that in our studies involving knockdown of *Sec11a*, which is thought to specifically function as a component of the SPC, we observe a reduction in insulin storage and secretion. This finding suggests that defective SPC function alone can lead to the phenotypes we observe upon *Nnat* deficiency in vitro and in vivo.

Previous studies have also indicated that neuronatin may play a role in the central nervous system regulation of metabolism, and in particular body weight, as its expression in specific hypothalamic regions is regulated by leptin and nutrients (12, 13). Furthermore, rare single-nucleotide polymorphisms at the human *NNAT* locus are associated with extreme childhood obesity (17). The lack of marked body weight, feeding, or energy expenditure phenotypes in *Nnat*-null mice, plus preserved sensitivity to exogenous leptin, suggests that neuronatin does not play a major role in these aspects of energy metabolism.

In summary, we demonstrate an unexpected role for neuronatin in the control of early preproinsulin SP cleavage, which leads to marked deficits in insulin storage and secretion, thus defining a previously unrecognized site of nutrient-sensitive control of β cell function and whole-animal glucose homeostasis. Importantly, the requirement for neuronatin in SP cleavage, and the expression of this protein in a range of neuronal, adipose, and endocrine cell types involved in the regulation of metabolism and growth, suggest additional roles in neuroendocrine cell secretion and the regulation of energy homeostasis. Future studies will be required to explore these possibilities.

## Methods

### Reagents.

Mouse monoclonal antibodies against insulin (clone K36AC10, I2018, for immunostaining of paraffin sections), FLAG (M2, F1804), β-tubulin (DM1A, T9026), and glucagon (K79bB10, G2654) were from Sigma-Aldrich. Rabbit polyclonal antibodies against glucagon (ab92517), NNAT (ab27266), VAPB (ab72470), and ribophorin 1 (ab198508) and mouse monoclonal antibodies against PDI (RL90, ab2792) were from Abcam. Mouse anti-insulin (L6B10, 8138, for immunoblotting/immunofluorescence), anti–c-Myc (9B11, 2276), and anti-IRE1α (14C10, 3294) were purchased from Cell Signaling. MG132 was purchased from Sigma-Aldrich.

### Animal models.

Experiments involving animals were designed and reported following Animal Research: Reporting of *In Vivo* Experiments (ARRIVE) guidelines. Power calculations for numbers of mice for each experiment were based on reported or known effect sizes and variation, in order to maximize chances of meaningful results without the unnecessary use of experimental animals. Where possible, investigators were blinded to the genotype of both study animals and tissue/blood samples. For experiments involving treatments, mice were randomized by genotype for study groups, or a crossover design was used where indicated. All metabolic studies were replicated in at least 2 independent cohorts.

A floxed *Nnat* allele was created by homologous recombination in embryonic stem (ES) cells. *LoxP* sites were introduced flanking exon 1 of the *Nnat* gene, with a *neomycin* cassette flanked by FRT sites used as a selectable marker (GenOway). 129S2/SvPas ES cells were transfected with linearized targeting vector JSC1-HR by electroporation, and DNA from clones screened by Southern blotting and PCR. ES cells were injected into blastocysts, which were implanted into pseudopregnant females and chimeric male mice screened for germline transmission. *Flp*-mediated excision to delete the *neomycin* selection cassette (Neo) was performed in vivo by breeding of mutant animals with ubiquitous *Flp* recombinase–expressing deleter mice, to produce a floxed *Nnat* allele (*Nnat^+/fl^*). Mice with constitutive deletion of *Nnat* were then created by intercrossing of the *Nnat^+/fl^* line with a *Cre* recombinase–expressing germline deleter strain in order to generate a null version of this allele (*Nnat^+/–^*). Both of these mouse lines were backcrossed in our transgenic animal facility more than 8 times onto C57BL/6J or 129S2/Sv backgrounds before intercrossing of mutant animals for generation of experimental cohorts. The presence of the mutant allele was determined by PCR analysis of DNA from ear tissue biopsies. A common upstream primer (5′-TGGTCTACTTCTCCATAAAGCTCGCTCC-3′) and primers specific for the WT allele (5′-GATCTTTTCTGACTGTTGGTTCCCGC-3′) and a region downstream of the excised sequence (5′-AAGGGGCATTTTTTTCTCTAGTGTGTTTCC-3′) were used for amplification. To delete *Nnat* specifically in pancreatic β cells, *Nnat^+/fl^* mice were crossed with transgenic mice expressing *Cre* recombinase at the *Ins1* locus (23) to generate mice with conditional deletion of *Nnat* in β cells.

Experimental cohorts of group-housed, male mutant mice and their control littermates were maintained on a 12-hour light/12-hour dark cycle with free access to water and standard mouse chow (RM3, Special Diet Services) and housed in specific pathogen–free barrier facilities. All animal work was carried out in accordance with the UK Animals (Scientific Procedures) Act (1986). High-fat diet (D12451; 45% energy from fat, 35% energy from carbohydrate) and high-sugar/high-fat Western diet (D12079B; 40% energy from fat, 43% energy from carbohydrate) were both purchased from Research Diets.

### Metabolic analysis.

Measurement of body weights, tail vein blood collection, and determination of blood glucose levels using a Contour glucometer (Bayer) were all performed as previously described (46). Intraperitoneal glucose (2 g/kg) and insulin (0.75 IU/kg) tolerance tests as well as intraperitoneal GSIS (3 g/kg) were performed as previously described (47) with blood collection at times indicated in the figures. Assessment of food intake and leptin sensitivity was performed in singly housed mice for 3 consecutive experimental days as previously described (46) using 1.5 mg/kg recombinant mouse leptin (R&D Systems). Levels of mature insulin (no cross-reactivity with proinsulins) were determined by ELISA (Crystal Chem), and levels of proinsulin and glucagon were determined by ELISA (Mercodia).

### Primary islet isolation and GSIS.

For islet isolation, the pancreas was injected with Liberase TM (Roche), digested at 37°C, and islets purified using a Histopaque gradient (Sigma-Aldrich), and handpicked as previously described (48). For secretion studies, isolated islets were cultured overnight in RPMI supplemented with 10% FBS. Following a 30-minute preincubation in Krebs-HEPES-bicarbonate buffer (KHB; 140 mM NaCl, 3.6 mM KCl, 0.5 mM NaH_2_PO_4_, 0.2 mM MgSO_4_, 1.5 mM CaCl_2_, 10 mM HEPES, 25 mM NaHCO_3_) with 3 mM glucose, GSIS was assessed by static incubation of 6 size-matched islets in KHB with 3 mM or 16.7 mM glucose for 30 minutes at 37°C. Mature insulin secreted into the media and total mature insulin content of the islets were assayed by ELISA (Crystal Chem; no cross-reactivity with proinsulins).

### Calcium imaging.

Intracellular free Ca^2+^ was imaged in KHB saturated with 95% O_2_/5% CO_2_ and adjusted to pH 7.4, with additions as stated in the figures. Islets were incubated at 37°C for 45 minutes in KHB containing 10 μM Fluo-2-AM (Cambridge Biosciences). Islets were then transferred in a perfusion chamber, mounted on a Zeiss Axiovert confocal microscope, and continuously perfused at 34°C–36°C. Fluo-2 was excited with a 491-nm laser line and emitted light filtered at 525/50 nm. Images were acquired with a Hamamatsu ImagEM camera, and Volocity software (PerkinElmer) was used for data capture. Traces are presented as normalized intensity over time (*F*/*F*_0_) (49).

### Transmission electron microscopy.

Isolated islets were fixed in 4% paraformaldehyde (PFA)/0.2% glutaraldehyde in PBS at 4°C overnight immediately upon extraction. Samples were washed 3 times with cacodylate buffer (Electron Microscopy Sciences [EMS]) and osmicated with osmium tetroxide in 2% (wt/vol) cacodylate buffer for 1 hour. Samples were then washed 5 times with deionized water and dehydrated through a graded ethanol series. After dehydration, samples were infiltrated with Epon Resin (EMS) diluted in ethanol at 3:1, 2:1, and 1:1 for 1 hour each, and then overnight at 1:2. The solution was then replaced with pure resin, which was changed twice in the first 12 hours and then allowed to infiltrate again overnight. Samples were immediately placed in an oven at 60°C and left to cure overnight. Curetted resin blocks were sectioned at 100 nm using an ultramicrotome (Leica) and immediately placed on copper grids. These grids were imaged in a transmission electron microscope at 80 kV (JEOL 2000FX TEM).

### Histological techniques and immunofluorescence.

Dissected pancreata were fixed in Bouin’s solution, embedded in paraffin, and cut into 5-μm sections. Insulin (mouse monoclonal, Sigma-Aldrich) and glucagon (rabbit polyclonal, Abcam) immunostaining and morphometric analysis were performed blindly as previously described using a DMRB Fluorescence microscope (Leica) (46). Dispersed islets and INS1E cells grown overnight on poly-l-lysine coverslips were fixed in 4% PFA, permeabilized, and immunostained with primary (listed in the *Reagents* section) and secondary antibodies (Alexa Fluor 488 and 594, Invitrogen). Cells were mounted on glass slides and imaged using a TCS SP5 II Confocal microscope (Leica).

### Immunoprecipitation and mass spectrometry.

MIN6 cells (24) grown in standard culture conditions (DMEM, 10% FBS, 2 mM l-glutamine, 50 μM β-mercaptoethanol, 37°C, 5% CO_2_) were homogenized in protein lysis buffer (50 mM Tris-HCl, pH 7.5, 150 mM NaCl, 1% Triton X-100, 1 mM EDTA with protease inhibitors [Complete Mini, Roche]), clarified, and normalized for protein content. Supernatants were incubated with protein A-agarose beads (Calbiochem) and antibodies against NNAT (rabbit polyclonal, Abcam) or rabbit IgG (control immunoprecipitate) rotating at 4°C overnight. Beads were washed 4 times with lysis buffer, boiled in Laemmli buffer, and run on SDS-PAGE, and gels were stained with Coomassie (SimplyBlue, Invitrogen). Each lane was excised into bands and placed into separate protein LoBind (Eppendorf) Eppendorf tubes for proteomic analysis by mass spectrometry. Coomassie was removed from bands by incubation with 50 mM ammonium bicarbonate, 10% acetonitrile for 30 minutes at room temperature and gel pieces dehydrated for 10 minutes. Disulfide bonds were reduced using 10 mM DTT and free thiol groups alkylated with 55 mM iodoacetamide. Modified porcine trypsin (Proteomics Grade, Sigma-Aldrich) was added to each gel piece to a final amount of 0.5 μg and incubated overnight at 37°C, with tryptic digestion halted by addition of 5% formic acid. Peptide-containing solutions were dried in a vacuum centrifuge, before peptides were solubilized in 30 μl of 0.1% trifluoroacetic acid (TFA) and placed in autosampler vials ready for liquid chromatography/mass spectrometry injection.

Peptides were separated using an UltiMate 3000 nano liquid chromatography system (Thermo Fisher Scientific) before mass spectrometric analysis. Five microliters of sample was loaded onto a trap column (Acclaim Pepmap 100; 100 μm × 2 cm, C18) at 8 μl/min in 2% acetonitrile, 0.1% TFA. Peptides were then eluted online to an analytical column (Acclaim Pepmap RSLC; 75 μm × 25 cm, C18) and separated using a ramped gradient. Eluted peptides were analyzed by an LTQ Velos Orbitrap mass spectrometer operating in positive polarity data-dependent acquisition mode. Ions for dissociation were determined from an initial 15,000-resolution mass spectrometric survey scan (event 1) followed by collision-induced dissociation (CID) of the top 10 most abundant ions (500 count threshold). CID conditions were default charge state 2, 2.0 *m*/*z* isolation width, normalized collision energy 35.0, activation *Q* value 0.25, activation time 10 milliseconds, lock mass value 445.120030 *m*/*z*. Progenesis QI for Proteomics (Waters Corp.) software was used to align all raw data files, perform peak picking, and produce features for export as.mgf files. Mascot and Mascot Daemon v2.5.1 (Matrix Science) were used to search.mgf files against the Swiss-Prot *Mus musculus* database (https://www.uniprot.org/) (downloaded November 26, 2014, containing 16,686 sequences). A reversed decoy was implemented by Mascot, and results were filtered to 1% false discovery rate. Search parameters included: 10 ppm MS1 tolerance, 600 milli mass unit MS2 tolerance, maximum of 2 missed cleavages, fixed modification of cysteine carbamidomethylation, and variable modifications of methionine oxidation and protein N-terminal acetylation. Resulting Extensible Markup Language (XML) files were returned to Progenesis and proteins filtered for those containing at least 1 unique peptide and identified in all 3 biological replicates. Intensity normalization was performed by Progenesis, and proteins were ranked by fold change increase in abundance compared with control immunoprecipitates.

For validation experiments in HEK293T cells (cultured in DMEM, 10% FBS, 2 mM l-glutamine, 37°C, 5% CO_2_), cells were transfected with plasmids expressing C-terminal c-Myc–tagged SEC11A (Origene) and C-terminal FLAG-tagged mouse NNAT in pcDNA3.1 (BamHI/XhoI sites) using Lipofectamine 2000. Forty-eight hours later, transfected cells were lysed, clarified, normalized as above, and immunoprecipitated using anti–c-Myc agarose resin (Pierce) with Western blotting performed using antibodies against c-Myc (mouse monoclonal, Cell Signaling) and FLAG (mouse monoclonal, Sigma-Aldrich).

### siRNA knockdown.

For siRNA silencing, INS1E cells (50) (cultured in RPMI, 10% FBS, 2 mM l-glutamine, 50 μM β-mercaptoethanol, 37°C, 5% CO_2_) were transfected with 50 nM of Silencer Select siRNAs (*Nnat*, s137247; *Sec11a*, s80747; both Ambion) using Dharmafect 1 reagent (Dharmacon). After 24 hours, cells were incubated overnight in cell culture medium with 3 mM glucose. For GSIS the following day, cells were incubated for 30 minutes in KHB with 3 mM glucose at 37°C and then with 3 mM glucose or 25 mM glucose for a further 30 minutes at 37°C. Insulin secreted into the media and total cellular insulin content were assayed by ELISA (Crystal Chem).

### Pulse-chase analysis.

Radiolabeling was performed in INS1E cells with transient transfection of C-terminal c-Myc–tagged mouse *Preproinsulin2* (Origene) and siRNAs. Forty-eight hours after transfection, cells were pulse-labeled with ^35^S-methionine/cysteine (PerkinElmer) for 15 minutes and chased with unlabeled media where indicated, essentially as previously described (40, 41). Cells were homogenized in protein lysis buffer as above and centrifuged at 16,000 *g* for 10 minutes at 4°C. Cell lysates and chase media were immunoprecipitated using c-Myc agarose resin (Pierce), and analyzed by SDS-PAGE and standard autoradiography techniques including enhancement with Amplify reagent (GE Healthcare).

### ER targeting assay.

For assessment of preproprotein targeting to the ER, INS1E cells were transfected with preproinsulin 2 and siRNAs as above with the inclusion of a vector expressing c-Myc–tagged mouse GAPDH (Origene) and radiolabeled for 15 minutes. Cells were then washed and resuspended in 50 μl of 50 mM HEPES, 150 mM NaCl, 2 mM CaCl_2_, pH 7.5, with protease inhibitors with or without 0.01% digitonin for 10 minutes on ice. Cells were centrifuged at 16,000 *g* for 10 minutes at 4°C, after which 450 μl of protein lysis buffer (1% Triton X-100, as above) was transferred to the supernatant and the pellet lysed in 500 μl protein lysis buffer. Both supernatant and pellet were immunoprecipitated with c-Myc agarose resin and analyzed by autoradiography as above, all as described previously (40, 41), and with 3 independent experiments performed.

### Assays in partially permeabilized cells.

For membrane orientation of NNAT in INS1E, cells grown on poly-l-lysine coverslips were fixed with 4% PFA, permeabilized with either 0.01% digitonin or Triton X-100, and immunostained with antibodies against NNAT (rabbit polyclonal, Abcam) or PDI (mouse monoclonal, Abcam). Images were acquired using a TCS SP5 II Confocal microscope (Leica), all as previously described (40). To assess preproinsulin translocation across the ER membrane, INS1E cells were grown on poly-l-lysine coverslips, with siRNA-mediated knockdown of *Nnat* for 48 hours. Cells were pretreated with 10 μΜ MG132 for 4 hours before fixation and permeabilized with either digitonin or Triton X-100 as above, and immunostaining was performed using antibodies against preproinsulin/mature insulin (Cell Signaling), NNAT, and PDI. Both of these experiments were performed at least 3 times independently, and representative images shown are maximum projections from 3-step *Z*-stacks 0.5 μm apart.

### Cellular membrane preparation.

INS1E cells were homogenized in a hypotonic buffer (50 mM Tris-HCl, pH 7.5, 50 mM mannitol, 1 mM EDTA with protease inhibitors) and cleared by centrifugation at 1,000 *g* for 10 minutes at 4°C. The supernatant was layered on a mannitol cushion (same buffer as above but 300 mM mannitol) and ultracentrifuged (Optima TLX Ultracentrifuge, Beckman Coulter) at 60,000 *g* for 60 minutes at 4°C. Both the crude membrane pellet and the supernatant were resuspended in Laemmli buffer and boiled and proteins analyzed by Western blotting using antibodies against NNAT (rabbit polyclonal, Abcam), VAPB (rabbit polyclonal, Abcam), and β-tubulin (mouse monoclonal, Sigma-Aldrich).

### Expression analysis.

Snap-frozen tissues or cells were homogenized directly into Trizol reagent for RNA analysis or lysis buffer (50 mM Tris-HCl, pH 7.5, 150 mM NaCl, 1% Triton X-100, 1 mM EDTA with protease inhibitors [Complete Mini, Roche]) for protein work. mRNA was extracted using RNeasy kits (Qiagen) and normalized for cDNA synthesis, and expression was assessed by quantitative RT-PCR using TaqMan reagents on a 7900HT Real Time PCR cycler (all Applied Biosystems). A complete list of probes used for quantitative RT-PCR is available in Supplemental Table 2. Protein expression was analyzed by Western blotting from clarified lysates normalized for protein content by BCA method (Bio-Rad), all as previously described (46). A complete list of primary antibodies can be found in the *Reagents* section.

### Signal peptidase assay.

Cell-free cleavage of SP from preprohormones in vitro has been described previously (28, 29). Briefly, cDNAs for *Preproinsulin2* and subunit components of the SPC (*Sec11a* and *Spcs3*), all with a C-terminal c-Myc tag (purchased from Origene), as well as C-terminal FLAG-tagged full-length *Nnat* and untagged *Gfp* were PCR-amplified and ligated into the MscI/SalI sites of the pT7CFE1.CHis vector. Stop codons were included after each tag to prevent expression of the endogenous His tag. Proteins were translated from these constructs in vitro using a human (HeLa-based) in vitro translation kit (Pierce) by incubation at 30°C for 90 minutes. Where indicated, canine pancreatic microsomes (Promega) were included in the reaction. A standard concentration of pancreatic microsomes was used per reaction as a source of SPC, which generated partial (~50%) processing of the total amount of preproinsulin, but allowed a significant proportion of unprocessed preproinsulin in order to determine augmenting effects of exogenous NNAT on these processing dynamics. Samples from 3 independent experiments were boiled in Laemmli buffer and the abundance of proteins in these reactions determined using Western blotting with anti–c-Myc and -FLAG antibodies.

### NNAT membrane topology.

For orientation of NNAT on the microsomal membrane, 90-minute translations expressing NNAT, SPCS3, or SEC11A were adjusted to a final concentration of 2 mM CaCl_2_, and/or 10 μg/ml proteinase K, and/or 1% Triton X-100 where indicated, and transferred to ice for 30 minutes. Addition of Triton X-100 detergent to disrupt microsomes served as a positive control for proteinase K activity. Proteolysis was terminated by addition of PMSF, and samples from 3 independent experiments were boiled in Laemmli buffer before analysis by Western blotting using antibodies against c-Myc and FLAG, essentially as previously described (31).

### Statistics.

Assessment of normal distribution (D’Agostino-Pearson normality test) and also statistical significance between groups was determined using GraphPad Prism 7 by 2-tailed Student’s *t* test or ANOVA, or Mann-Whitney *U* or Kruskal-Wallis tests as the nonparametric equivalent. Bonferroni (or Dunn’s for nonparametric) and Geisser-Greenhouse post hoc tests were performed to correct for multiple comparisons and repeated measures, respectively, where appropriate. A probability of error less than 5% was considered significant (i.e., *P* < 0.05). Statistical information for experiments (data representation, *P* values, and *n* numbers) can be found in the figure legends. In all panels, data are represented as mean ± SEM.

### Study approval.

All of the animal procedures conformed to the UK Animals (Scientific Procedures) Act 1986 as well as being approved by Imperial College Animal Welfare and Ethical Review Body and by the UK Home Office.

## Author contributions

SJM, GAR, JS, and DJW designed the research. SJM, GDSX, AIC, SB, PC, SMAP, EEI, AM, PF, JKC, FP, and M. Latreille performed experiments. WRT and JF contributed new reagents/analytical tools. SJM, RMJ, M. Latreille, M. Liu, GAR, JS, and DJW drafted and/or wrote the manuscript. MC, GAR, JS, and DJW provided funding. GAR, JS, and DJW supervised the work.

## Supplementary Material

Supplemental data

Supplemental Table 1

## Version 1 06/04/2018

In-Press Preview

## Version 2 07/09/2018

Electronic publication

## Version 3 08/01/2018

Print issue publication
