# Supplementary material for: Neuronatin regulates pancreatic β cell insulin content and secretion
Source: J Clin Invest. 2018 Jul 9;128(8):3369–81. doi: 10.1172/JCI120115 (PMC6063487; doi:10.1172/JCI120115)
Supplement: Supplemental data [file jci-128-120115-s103.pdf]

Supplemental data

Figures and figure legends

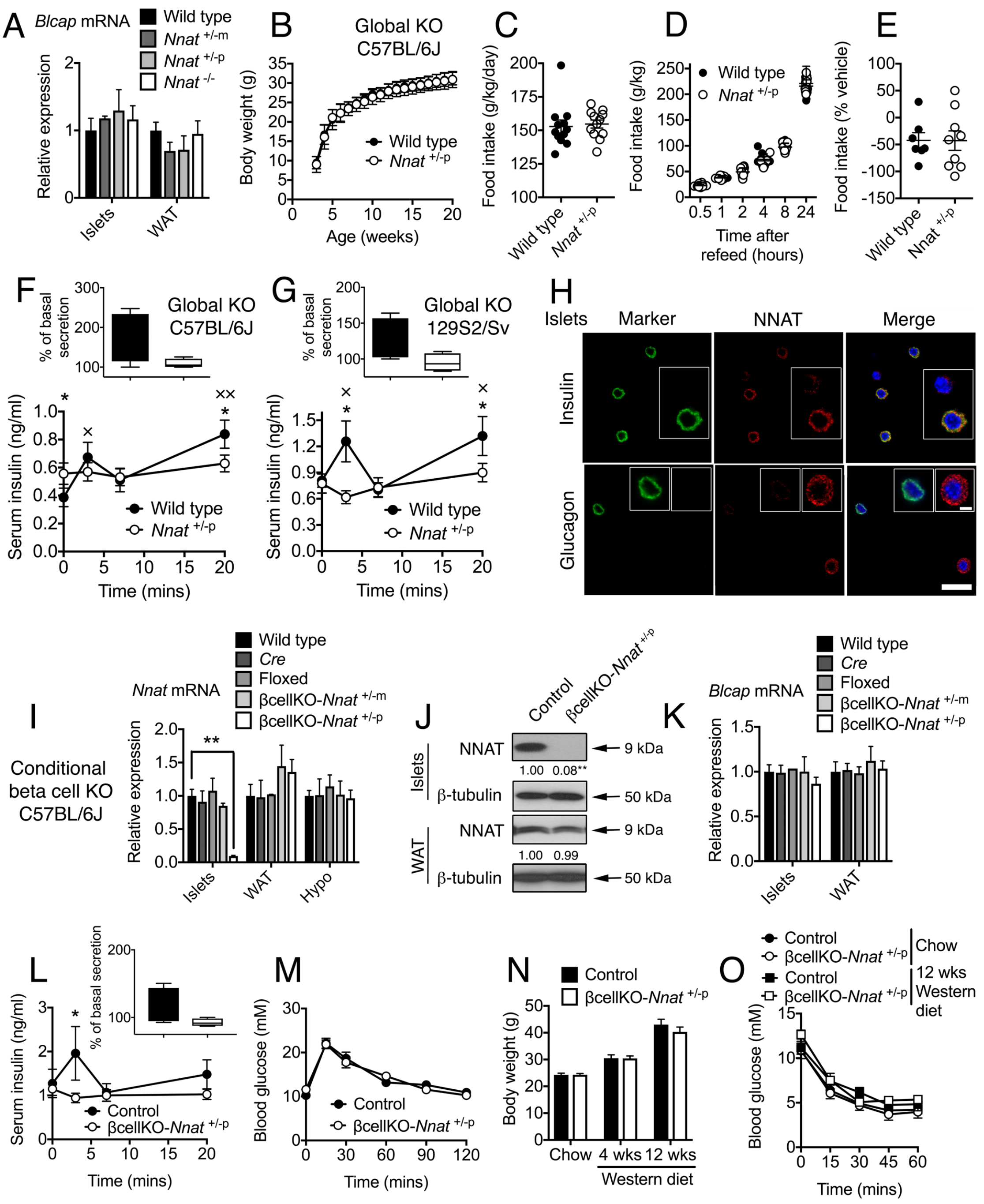

Supplemental Figure 1. Metabolic profiling of *Nnat* deficient mice. (A) RT-PCR analysis of *Blcap* mRNA expression in isolated islets and WAT of wild type, heterozygous *Nnat*$^{+/-m}$ (maternal deletion), heterozygous *Nnat*$^{+/-p}$ (paternal deletion) and

homozygous *Nnat*$^{-/-}$ mice on C57BL/6J background. *Hprt* and *Cyclophilin A* mRNA was used as an internal control for islets and WAT respectively and data is compared to wild type mice (n = 6 animals per genotype). (B) Body weights of wild type and *Nnat*$^{+/-p}$ mice on C57BL/6J background (n = 19 and 23 for wild type and *Nnat*$^{+/-p}$ mice respectively). (C, D) *Ad libitum* feeding (C) and feeding following an overnight fast (D) in 10 week old male wild type and *Nnat*$^{+/-p}$ mice (C57BL/6J) (n = 12 animals per genotype, 2 independent cohorts). (E) Food intake alterations (percentage change) in response to exogenous leptin compared to internal saline crossover control in 10 week old male wild type and *Nnat*$^{+/-p}$ mice (C57BL/6J) (n = 7 and 9 for wild type and *Nnat*$^{+/-p}$ mice respectively, 2 independent cohorts). (F, G) Measurement of insulin secretion in vivo in response to i.p. glucose in 10 week old male *Nnat*$^{+/-p}$ mice on a C57BL/6J (F) and a 129S2/Sv (G) background (n = 10 animals per genotype, minimum 2 independent cohorts). Inset shows box and whiskers plot of the same data plotted as percentage insulin secretion across all time points compared with basal insulin values (at T = 0), demonstrating the dispersion of insulin secretion increase in wild type animals across all time points that was completely lacking in *Nnat*$^{+/-p}$ mice (x - $p < 0.05$ and xx - $p < 0.01$, indicating statistically significant increases in secretion in wild type mice at each time point compared with basal insulin values, ANOVA with repeated measures). (H) Immunofluorescent staining of dispersed islet cells using antibodies against NNAT (red) and markers of beta (Insulin, green) and alpha (Glucagon, green) cells assessed by confocal microscopy. Nuclei were visualized with DAPI. Scale bar = 25 μm. Inset shows individual cells with 2x digital zoom, scale bar = 5 μm. (I, J) RT-PCR and representative Western blot analysis of *Nnat* expression in isolated islets, WAT and Hypothalamus (Hypo) in wild type, *Ins1Cre* positive (*Cre*), *Nnat*$^{+/flox}$ (Floxed), *Ins1Cre* positive *Nnat*$^{+/flox}$ from maternal allele (βcellKO-*Nnat*$^{+/-m}$) and *Ins1Cre* positive *Nnat*$^{+/flox}$

from paternal allele (βcellKO-*Nnat*$^{+/-p}$) mice on C57BL/6J background. *Hprt* (Islets and Hypo) and *Cyclophilin A* (WAT) mRNA were used as internal controls, and data is compared to wild type mice. β-tubulin was used as a protein loading control (n = 8 and n = 4 animals per genotype for RT-PCR and Western blot respectively, one-way ANOVA and Student t test, respectively). (K) RT-PCR analysis of *Blcap* mRNA expression in isolated islets and WAT in the same mice as I. (L) Measurement of insulin secretion in vivo in response to i.p. glucose in 22 week old male βcellKO-*Nnat*$^{+/-p}$ vs control mice on C57BL/6J background fed Western diet for 12 weeks (n = 7 animals per genotype, ANOVA with repeated measures). Inset shows box and whiskers plot of the same data plotted as percentage insulin secretion across all time points compared with basal insulin values (at T = 0). (M) Glucose tolerance (overnight fast) in 10 week old chow-fed male wild type and βcellKO-*Nnat*$^{+/-p}$ mice (C57BL/6J) (n = 5). (N, O) Body weights (N) and insulin sensitivity (O) in 14 week old chow and Western diet fed either for 4 weeks (14 weeks old) or 12 weeks (22 weeks old) male wild type and βcellKO-*Nnat*$^{+/-p}$ mice (C57BL/6J) (n = 7 and 10 mice for chow and Western diet per genotype respectively, minimum 2 independent cohorts). (* $p < 0.05$, ** $p < 0.01$).

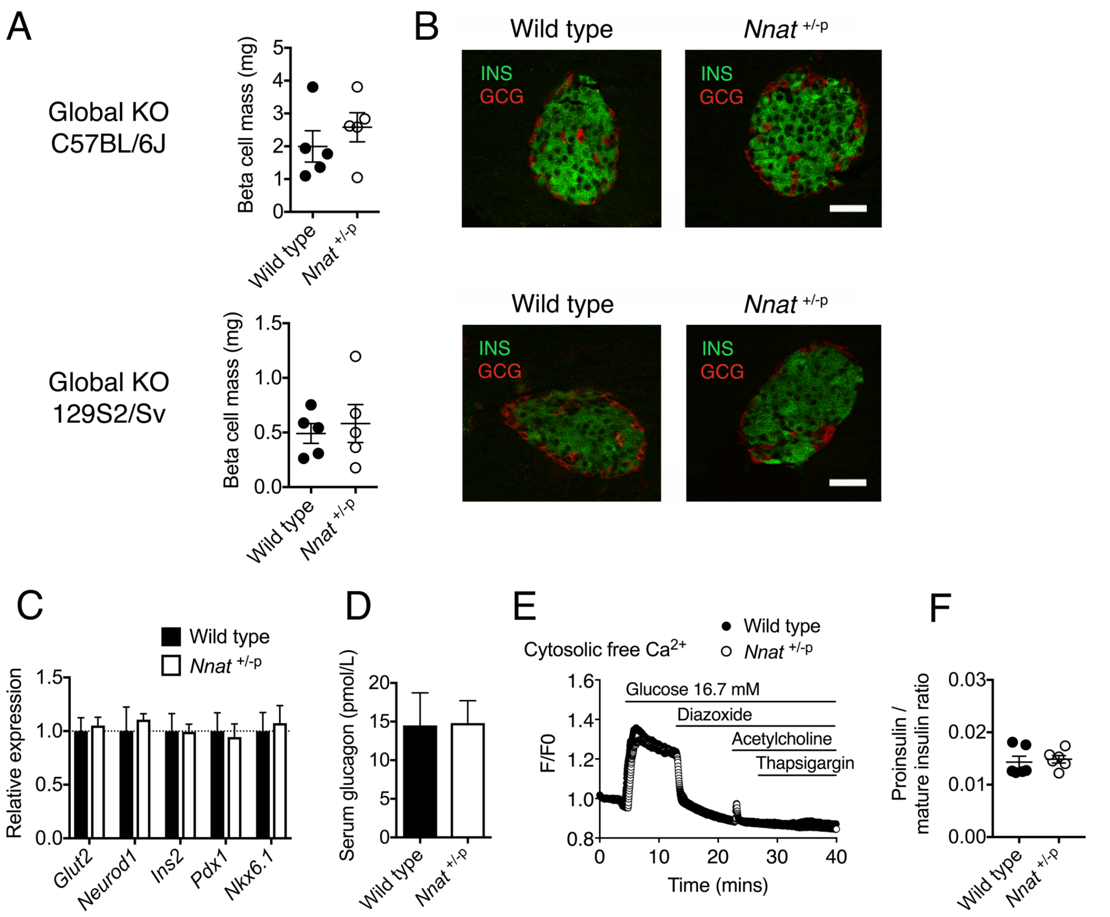

Supplemental Figure 2. Assessment of islet function upon *Nnat* deletion. (A, B) Morphometric analysis of β-cell mass (A) in pancreatic sections from 10 week old wild type and *Nnat*$^{+/-p}$ mice on a C57BL/6J and a 129S2/Sv background (*n* = 5 animals per genotype) with representative immunofluorescent images (B) showing staining of insulin (INS, green) and glucagon (GCG, red). Scale bar = 25 μm. (C) Quantitative RT-PCR analysis of mRNAs encoding key beta cell genes in isolated islets of 10 week old wild type and *Nnat*$^{+/-p}$ mice. *Hprt* mRNA expression was used as an internal control and data is represented relative to wild type mice (*n* = 6 animals per genotype). (D) Glucagon levels in serum of fasted 10 week old wild type and *Nnat*$^{+/-p}$ mice (C57BL/6J) (*n* = 9 mice per genotype). (E) Ca^{2+}-bound Fluo-2 fluorescence in response to high glucose (16.7 mM) and other agents inprimary islets expressed as normalised intensity over time (F / F0) (*n* = 18 islets total from 6 mice per genotype, 2 independent

experiments). (F) Proinsulin to mature insulin ratio (n = 6 animals per group) was quantified and calculated in isolated islets from wild type and $Nnat^{+/-p}$ mice.

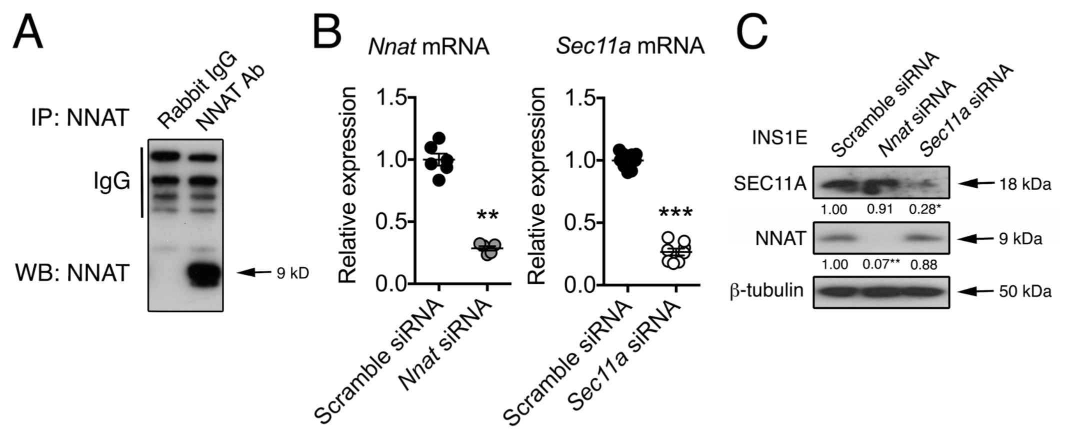

Supplemental Figure 3. NNAT immunoprecipitation and knockdown. (A) MIN6 cell lysates were immunoprecipitated with antibodies against NNAT (NNAT Ab) or rabbit IgG as a control, with Western blotting (representative blot shown) used to check the presence of endogenous NNAT in immunoprecipitates. (B) Quantitative RT-PCR analysis of mRNA encoding *Nnat* and *Sec11a* in INS1E cells transfected with control (Scramble), *Nnat* or *Sec11a* siRNA. *Hprt* expression was used as an internal control with data compared to scramble controls (n = 6 and 9 independent cultures per group for *Nnat* and *Sec11a* respectively, 3 independent experiments, both Mann-Whitney U test). (C) INS1E cells with siRNA-mediated knockdown of *Nnat* and *Sec11a* as in B were analysed by Western blotting for expression of these proteins. β-tubulin was used as a loading control and a representative blot is shown (Kruskal-Wallis test). (* $p < 0.05$, ** $p < 0.01$, *** $p < 0.001$).

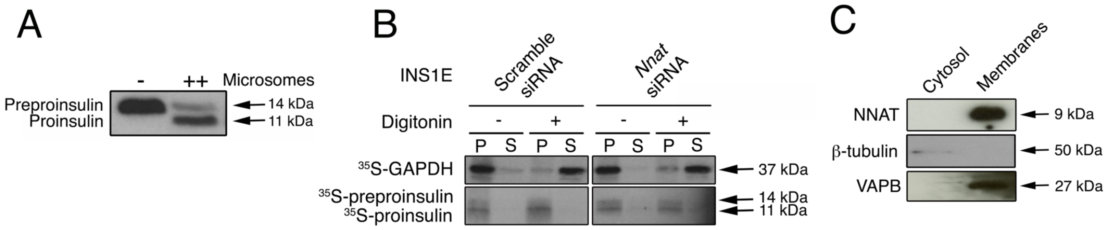

Supplemental Figure 4. In vitro peptidase assay and NNAT as a transmembrane protein. (A) Representative Western blotting analysis (3 independent experiments) of in vitro translated c-Myc tagged preproinsulin converted to proinsulin in the presence (++) or absence (-) of pancreatic microsomes (containing endogenous signal peptidase activity). (B) Targeting of c-Myc-tagged preproinsulin to the ER membrane assessed in INS1E with *Nnat* siRNA knockdown vs scramble siRNA control. Cells were pulse-labelled with ^{35}S-Cys/Met, treated with digitonin and the supernatant (S) and pellet (P) fractions immunoprecipitated with anti-c-Myc and analysed by autoradiography, with a representative image shown (*n* = 3 independent experiments per group/treatment. (C) A crude membrane fraction was prepared from clarified INS1E cell lysates with proteins from the high speed pellet (Membranes) and supernatant (Cytosol) fractions analysed by Western blotting using antibodies against NNAT, β-tubulin and VAPB.

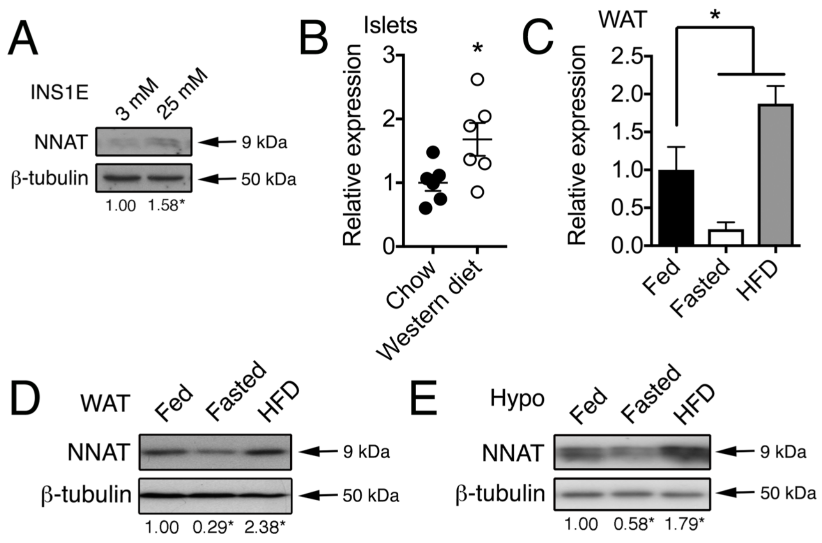

Supplemental Figure 5. NNAT regulation by glucose and the diet. (A) Representative Western blotting analysis of NNAT protein expression in INS1E cells cultured in low (3 mM) or high (25 mM) glucose conditions for 6 hours. Mean values for each condition are shown below the panel, compared with low glucose controls. β-tubulin was used as a loading control (*n* = 6 independent cultures per treatment, 2 independent experiments, Mann-Whitney U test). (B) Quantitative RT-PCR analysis of *Nnat* mRNA in isolated islets from 10 week old male wild type C57BL/6J mice fed Western diet for 72 hours. *Hprt* mRNA was used as an internal control and data is represented vs chow fed mice (*n* = 6 animals per group, Mann-Whitney U test). (C) Quantitative RT-PCR analysis of *Nnat* mRNA in WAT from 10 week old male wild type C57BL/6J mice fasted overnight (Fasted) or acutely fed HFD for 72 hours (HFD) vs chow fed controls (Fed). *Cyclophilin A* mRNA was used as an internal control (Kruskal-Wallis test). (D) Western blot analysis of NNAT protein expression in the same tissues with β-tubulin used as a loading control (*n* = 5 animals per group, Kruskal-Wallis test). (E) Representative Western blot analysis of NNAT protein expression in the hypothalamus (Hypo) of the same mice used in D. (* $p < 0.05$).

**Supplemental Table 2.**	
**List of plasmids and RT-PCR probes**	
**Plasmids**	**Source**
Expression in cell lines:	
pcDNA3.1-*Nnat*-FLAG	This paper
In vitro translation:	
pT7CFE1-*Preproinsulin2*-Myc	This paper
pT7CFE1-*Spcs3*-Myc	This paper
pT7CFE1-*Sec11a*-Myc	This paper
pT7CFE1-*Nnat*-FLAG	This paper
pT7CFE1-*Gfp*	Thermo Scientific (Pierce)
**Probes for RT-PCR (TaqMan, Applied Biosystems, mouse unless otherwise stated)**	
Nnat	Mm00440480_m1
Hprt	Mm00446968_m1
Cyclophilin A	Mm03302254_g1
Glut2	Mm00446224_m1
Neurod1	Mm01280117_m1
Ins2	Mm00731595_gH
Pdx1	Mm0435565_m1
Nkx6.1	Mm00454962_m1
Blcap	Mm00727119_s1
Atf6	Mm01295317_m1

Grp78	Mm00517691_m1
Chop	Mm00492097_m1
Xbp1s	Mm03464496_m1
Nnat (rat)	Rn00822063_m1
Sec11a (rat)	Rn00581878_m1
Hprt (rat)	Rn01527840_m1
